# Evaluation of the Equity of Urban Park Green Space Based on Population Data Spatialization: A Case Study of a Central Area of Wuhan, China

**DOI:** 10.3390/s19132929

**Published:** 2019-07-02

**Authors:** Chuandong Tan, Yuhan Tang, Xuefei Wu

**Affiliations:** Department of Landscape Architecture, College of Horticulture and Forestry, Huazhong Agricultural University, No. 1, Shizishan Street, Hongshan District, Wuhan 430070, China

**Keywords:** urban park green space, accessibility, equity, population data spatialization

## Abstract

To measure the equity of urban park green space, spatial matching between service supply and user group demand should be taken into consideration. However, if the demographic data, with the administrative division as the basic unit, are directly applied to characterize the spatial distribution of a user group, it may introduce inevitable deviation into the evaluation results due to the low-resolution nature and modifiable areal unit problem of such data. Taking the central area of Wuhan as an example, the population data spatialization method based on land use modeling was used to build a geographically weighted regression (GWR) model of land cover type and demographic data, and the spatial distribution of the population of the 150 m grid was obtained by inversion. Then, the equity of park green space in Wuhan central city was evaluated by population spatial data and network accessibility. The results showed that (1) the range of park green space in the central urban area of Wuhan was within a walking distance of 15 min, accounting for 25.8% of the total study area and covering 54.2% of the population in the study area; (2) the equity of park green space in Hongshan District was the worst; (3) and the use of population spatial data can measure equity on a more precise scale.

## 1. Introduction

As a key component of urban green infrastructure, the urban park green space can not only provide a series of ecosystem services such as stormwater management [1], heat regulation [2], and air purification [3], but also has many social-economic benefits, such as increasing neighborhood communication [4], providing natural leisure experience [5], promoting physical and mental health [6], and boosting real estate prices [7]. Therefore, the reasonable distribution of the park green space is of great significance for enhancing the overall well-being of urban dwellers [8]. However, with the acceleration of urbanization, park green space resources in most cities are hardly meeting the needs of rapid population expansion, and the phenomenon of the uneven distribution of park green spaces has received particular attention [9,10].

The study of the equity of urban park green spaces has gone through a development from quantitative equalization to spatial equity and finally formed social aspects. The early studies paid more attention to the quantity aspect of equity, such as the per capita green area, the green space rate, the greening coverage rate, and so on, which are still the official evaluation indexes in the current urban green space planning system in China. This method has some limitations, such as ignoring the spatial equity of green space layout, ecosystem service function, and resource sharing. With the concept of the accessibility and development of Geographic Information System (GIS) technology, increasingly, scholars have begun to pay attention to spatial distribution in the research into the equity of urban parks [11]. Accessibility analysis has also been widely used in spatial distribution studies of other important public service facilities in cities, such as health care services [12,13], shopping service centers [14] and sports facilities [15]. Accessibility only considers the opportunity and distance of individuals using park green space and thus is regarded as a kind of spatial equity. However, social equity places an emphasis on equal public services for all urban dwellers and focuses more on the satisfaction of the needs of special groups with limited mobility or with low socioeconomic status, including children, the elderly, low-income people, and ethnic minority people [10,16]. Therefore, in order to realize the social equity of the distribution of urban park green space, it is necessary to consider the spatial distribution of the needs of the users, thus reflecting the spatial match between the public services provided by the park and the users.

Evaluating the equity of public services should measure the spatial and quantitative relationship between user demand and object supply. When we evaluate urban park equity, the supply and demand are generally represented by park accessibility and population density, respectively [17]. Moreover, most of the relevant studies are carried out directly using census data [1,9,10] and assume that the population is evenly distributed within the statistical unit. However, there are some limitations to this assumption because of the spatial heterogeneity of population distribution. The direct use of population data based on administrative division statistics in micro and medium-scale urban research makes it difficult to meet the corresponding precision requirements, and there is a modifiable areal unit problem [18], which means the analysis results are affected by the selected analysis area unit. In order to improve the accuracy of the evaluation, some studies directly use building data or residential committee data [19,20,21] to characterize population distribution. However, such data is often difficult to obtain. Therefore, this study introduces a population data spatialization method to obtain a more accurate spatial distribution of population, thus exploring a more universal and finer spatial-scale evaluation method of park equity.

Consequently, the aim of this study is to evaluate the equity of urban park green spaces in a finer spatial scale by using the population spatialization data. We choose Wuhan as our case study and focus on distributional equity in terms of equal access to urban park green spaces across all social groups. By constructing a regression model between the land cover and the statistical population, the grid map of the high-precision population distribution of Wuhan central area is obtained, and the corresponding population demand is measured. Moreover, the accessibility analysis of the parks was evaluated quantitatively by using the ArcGIS Network analysis tools, and the spatial superposition analysis method is used to analyze the spatial equity of the urban park green spaces in the study area.

## 2. Study Area and Data Sources

### 2.1. Study Area

Located in the heart of China’s hinterland, as the capital of Hubei province, Wuhan is the central city of the country central part. The Yangtze River and the Han River meet in the center of Wuhan. Rich in water resources, Wuhan has 140 rivers with a length of more than 5 km. Also, Wuhan contains 273 types of reservoirs and 166 lakes, of which 38 are in the central city.

**Figure 1:**
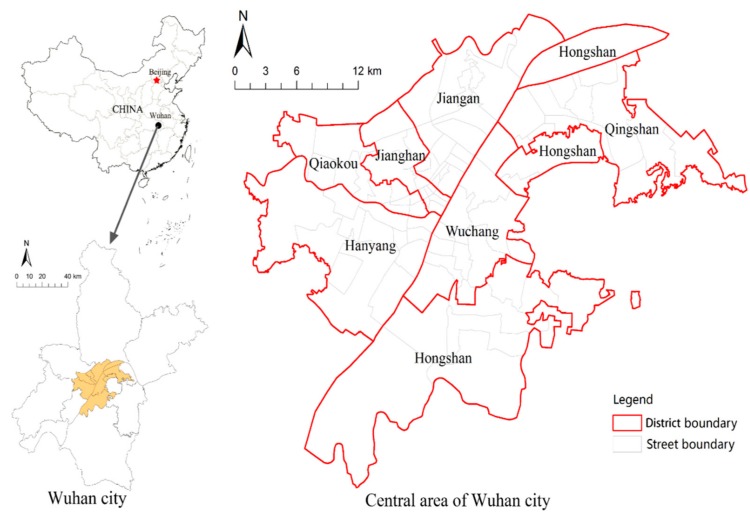
The location and administrative boundary of the study area.

Wuhan is under the jurisdiction of 13 administrative districts (including 7 central urban districts and 5 surrounding urban districts) and 4 development zones. The scope of this study includes the central areas of Wuhan City (Figure 1), which include Jiang’an, Jianghan, Qiaokou, Hanyang, Wuchang, Hongshan. and Qingshan. The plant area of WISCO (Wuhan Iron & Steel Co Ltd.) is excluded from the research scope due to the lack of data. Consisting of 89 streets, the study area is 628 km^2^ (including 28.9 km^2^ of green park) with a population of 5.726 million, and the average population density is 9118 per km^2^.

### 2.2. Data Sources and Preprocessing

**Figure 2:**
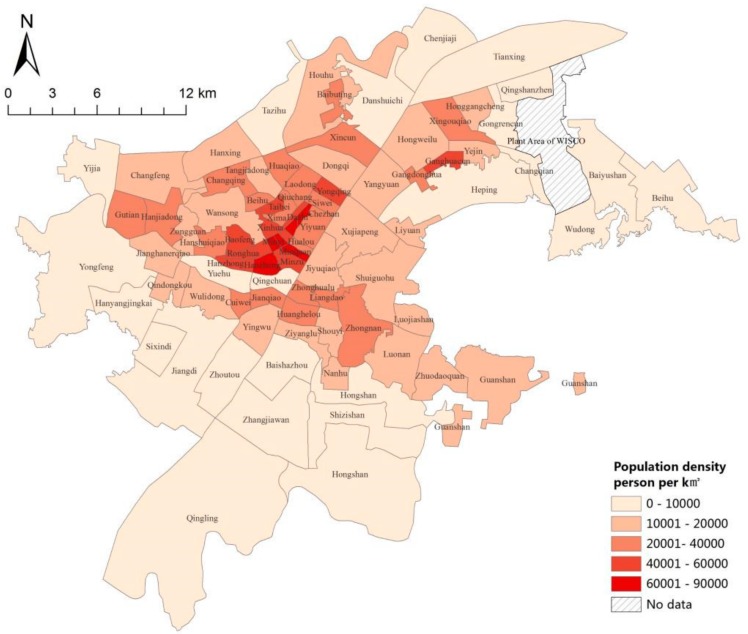
The street-level population density of the central area of Wuhan City in 2015.

**Figure 3:**
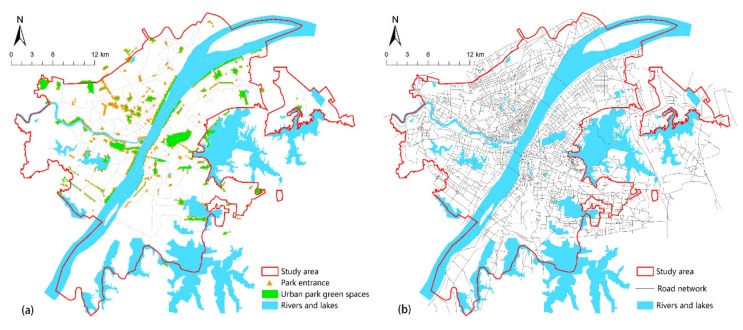
Urban park green space (**a**) and road network (**b**) in the central area of Wuhan City.

**Figure 4:**
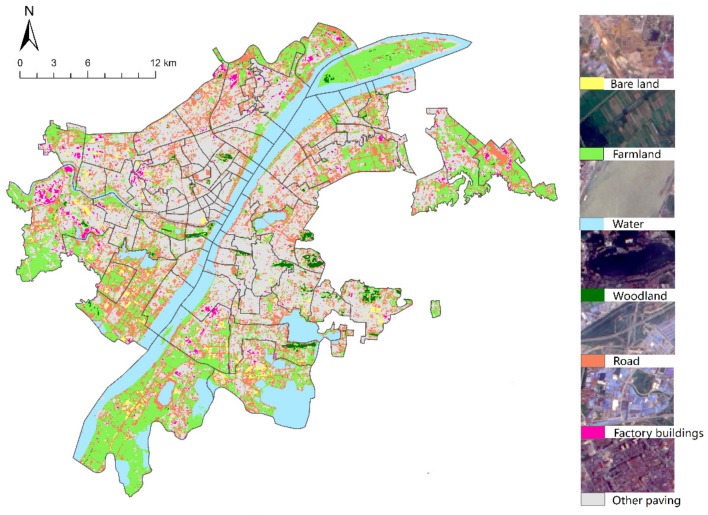
Land cover in the central area of Wuhan City.

(1) The population data are also derived from the statistical yearbook of Wuhan in 2016 (the date of the statistics is at the end of 2015), and the corresponding population density distribution of the block (Figure 2) is generated based on the administrative division data, which are based on the vectorization of raster maps of each district.

(2) The vector data of the green park (Figure 3a) required in this paper is derived from “the green space system planning of Wuhan”, taking the “boundary effect” into account, which will also include green parks in a 1500 m range outside the edge of the study area. The data is corrected by comparing current and historical Google Earth images, and the park entrance and exit location are determined by Baidu Map.
(3) The road network data in the region is interpreted from high-resolution Remote Sensing Satellite images. As shown in (Figure 3b), vector road network data of the study area were constructed after checking the topology. This data was used in the 2016 “Sponge City Sponge City Special Plan for Wuhan (Sponge City Sponge City Special Plan for Wuhan. http://www.wpdi.cn/project-3-i_11332.htm)”, this is a joint official planning project held by Wuhan planning and design institute and Wuhan natural resources and planning bureau. The road vector data were provided by Wuhan planning and design institute.
(4) The land cover data (Figure 4) were interpreted by landsat8 remote sensing image, which was dated 5 June 2016. The image was downloaded from the website of the China Geospatial Data Cloud (http://www.gscloud.cn/). Firstly, ENVI5.3 tool is used to preprocess the image, such as through radiometric calibration, atmospheric correction, fusion, cutting, and so on. Then, the maximum likelihood method is used to supervise and classify the images, which are divided into seven categories: Woodland, farmland, water, road, bare land, factory buildings, and other paving. We use high-precision Worldview 2 remote sensing images of the Wuchang district (dated 29 July 2016, with 1.8 m multispectral and 0.5 m panchromatic resolution) as ground truth samples for the classification accuracy evaluation. Ten regions of interest (ROIs) of each type of land cover were selected as evaluation samples by visual interpretation. Finally, we evaluated the classification accuracy using the confusion matrix method, and the results showed that the overall accuracy was 83.4192% and the Kappa coefficient was 0.8040.

## 3. Methods

### 3.1. Population Data Spatialization

The population data spatialization is based on the spatial distribution model of population and uses a certain calculation method to analyze the data and to present the implicit spatial information in order to simulate its geographical distribution. This method includes spatial interpolation [22], remote sensing spectral feature estimation [23], estimation model based on multi-source data [24,25,26], estimation model based on land use/land cover (LULC) [27], and so on. The theoretical basis of the spatial interpolation method is that the closer the spatial position is, the more likely it is to have similar eigenvalues. Thus, the statistical population based on administrative division is transformed into grid population data. The advantage of the interpolation method is that it is simple and feasible, and it can also eliminate the abrupt data on both sides of the administrative boundary line to a certain extent. The disadvantage is that only the conversion of the data presentation format is completed, and the ability to depict the real population distribution is weak [28]. The remote sensing spectral feature estimation method evaluates population distribution according to the relationship between remote sensing spectral characteristics and population data. Researchers such as Lo C. P. [29] studied the relationship between the gray value of thematic mapper (TM) images in different bands and urban population density. Because the population distribution is related to many factors, such as geographical location, land cover, road network convenience, water area, and economic development, etc. [19], some scholars have begun to use a variety of data as indicative factors related to population distribution. For example, Wang L. et al. [30] combined LULC data and night-light data and simulated the spatial distribution of population in China in 1990, 2000 and 2010. There are also many studies using point of interest (POI) data [31] and Volunteered Geographic Information (VGI) [32]. The estimation model based on LULC is the most widely used method for population spatial distribution simulation. The principle is based on establishing a multivariate statistical regression model based on LULC data and population distribution to estimate population distribution. However, due to the spatial heterogeneity of population distribution [33], compared with the Global Multivariate Statistical Regression model, the Geographical Weighted Regression (GWR) is more fitting to the performance of population distribution [34,35]. For example, Zhao Zhen et al. [35] used the GWR model to construct a spatial study of population data in southwest China. In this paper, by constructing a GWR model between LULC and demographic data, the regression coefficients are obtained to simulate the spatial distribution of population within the study area.

#### 3.1.1. Spatial Method of Population Data Based on Land Cover

This method assumes the same population density for the same land cover type. Firstly, the land cover data is obtained by using remote sensing image interpretation; then, the land cover data unit is taken as the explanatory variable, and the multivariate regression model is constructed with demographic data as the cause variable. The formula is as follows:

(1) $$p_{i}=\overset{n}{\underset{j=1}{\sum }}a_{j}\times S_{ij}+b$$

where *p_i_* is the statistical population of the *i*th block in the study area, *a_j_* is the population distribution coefficient under the *j* type land cover type, *S_ij_* is the area of the *j* land cover type in the *i* block, *n* represents the number of land cover types. According to the principle of “no land without population”, the constant *b* is 0.

According to Formula (1), we can simulate the predicted population value of each block, and then test the error according to the following formula:

(2) $$E=\frac{p_{i}^{\prime }-p_{i}}{p_{i}}\times 100\%$$

where *E* is the relative error, *p_i_* is the actual population count of block *i* in the study area, and $p_{i}^{\prime }$ is the predicted population in block *i*.

Since the model assumes that the distribution of population coefficients of the same land cover type is the same, there must be a deviation between the predicted population and the actual population. In order to ensure that the sum of the population data in the grid is consistent with the actual population sum, the adjustment method is used to revise the initial coefficients, and the process of rescaling is shown in the following equation:

(3) $$a_{ij}^{\prime }=\frac{p_{i}}{p_{i}^{\prime }}\times a_{j}$$

where $a_{ij}^{\prime }$ is the population distribution coefficient under category *j* of block *i* after being amended, *p_i_* is the actual population count of block *i* in the study area, and $p_{i}^{\prime }$ is the predicted population in block *i*.

A certain scale grid is constructed, and the land cover area in the grid is counted. On the basis of the correction coefficient, the population of the grid is inversely calculated to realize the rasterization of the population data. The grid demographics are shown in the following equation:

(4) $$pop_{ijk}=\overset{n}{\underset{j=1}{\sum }}a_{ij}^{\prime }\times s_{ijk}$$

where *pop_ijk_* is the population of the *k*th grid cell, $a_{ij}^{\prime }$ is the correction of population distribution coefficient under category *j* land cover type of block *i*, and *s_ijk_* is the area of the *j*th land cover type on the *k* grid cell in the *i* block.

#### 3.1.2. Geographical Weighted Regression (GWR)

In the geospatial analysis, the observed values of variables are generally obtained according to a given geographical unit as a sample unit, and with the change of geographical location, the relationship between variables will change, and the adjustment of the relationship between variables caused by the geographical location change is called spatial non-stationary adjustment. In order to solve the distortion of the analysis results caused by the non-smoothness of spatial data, the GWR model was proposed by Fotheringham on the basis of summarizing the previous studies on local regression and variable parameters [36]. The essence of the GWR model is to add a distance function as the weight on the basis of the traditional least squares method (OLS) regression model, so as to deal with the spatial non-stability of the data, and the related coefficients will change with the change of position. The model is in the following form:

(5) $$y_{i}=\beta_{0}\left(u_{i},v_{i}\right)+\overset{p}{\underset{k=1}{\sum }}\beta_{k}\left(u_{i},v_{i}\right)x_{ik}+\varepsilon_{i}$$

where (*u_i_, v_i_*) is the spatial coordinates of the sample point *i*, *β*_0_(*u_i_, v_i_*) is the intercept for location *I*, *β_k_* (*u_i_, v_i_*) is the local estimated coefficient of the independent variable *x_j_* at location *I*, and *ε_i_* is the error term.

In this paper, we use the ArcGIS10.2 GWR tool, taking demographic data as a dependent variable and land cover areas as independent variables. The adaptive method is used to calibrate the weighting function, and the optimal bandwidth is determined by the minimum Akaike information criterion (AIC).

The performance of OLS and GWR models was usually compared from two aspects: The predictive ability and the ability to address the spatial autocorrelation of variables [37,38]. We used the comparison model of the adjusted R^2^ and AIC. The higher the adjusted R^2^, the stronger the interpretation ability of variables, the smaller the AIC value, and the higher the fittest of the model. When the difference of the two models is greater than 3, it is considered that the model with a smaller AIC value is better. The Global Moran’s I index was calculated for the spatial autocorrelation; Moran’s I values range from −1 to 1. Values closer to 0 indicate that there is little or no spatial autocorrelation. If the Moran’s I index value is regular, it indicates a clustered trend, and if the Moran’s I index value is negative, it indicates a dispersed trend. If the distribution of the residuals obtained from the regression model has an obvious spatial autocorrelation, the assumption that residuals follow a random distribution is violated. That means the results of the model are not credible.

### 3.2. Network Accessibility Analysis

Network accessibility analysis is a method of calculating the service range of city parks under this cost value on the basis of a road network of the particular travel mode (by walking, cycling, bus or private car). Because it takes into account the actual routes and walking distances, network analysis can provide a more accurate measure of accessibility than using a simple buffering method [39,40]. A basic network consists mainly of centers, links, nodes, and cost. This study takes the real entrance and exit of the city park as the center, the city road as the link, while a node is an intersection between roads and the cost is expressed by the time spent on the road.

This study takes the time cost of residents walking to the park as a quantitative criterion for accessibility. With 1 m/s as the average walking speed and knowing the length of the road, the cost value of each link can be calculated, and the intersection cost value is set to be 0.5 min. According to people’s travel habits, a maximum of 15 min to reach the green park is regarded as sound accessibility.

### 3.3. Equity Evaluation

On the basis of the population spatial and network accessibility analysis, spatial superposition analysis is used to generate a low-accessibility range of the population spatial distribution map and to count the low-accessibility population at the street scale; these are the overall indicators to measure the equity evaluation of urban green parks.

## 4. Results

### 4.1. Spatial Population Data Based on Land Cover Type

#### 4.1.1. Performance Comparison of OLS and GWR Models

There is a close relationship between population distribution and land cover type. Using LANDSAT8 remote sensing image data, this study classifies the study area, which is divided into 7 categories: woodland, farmland, water, road, bare land, factory buildings, and other paving. Taking the block as the unit to count the area of each land type as a variable parameter, and then using SPSS to carry out Spearman correlation analysis with the population number respectively, the regression model could be built, with the results (Table 1) showing that the population distribution was significantly correlated with bare land, road, vegetation, factories, and paving.

**Figure 5:**
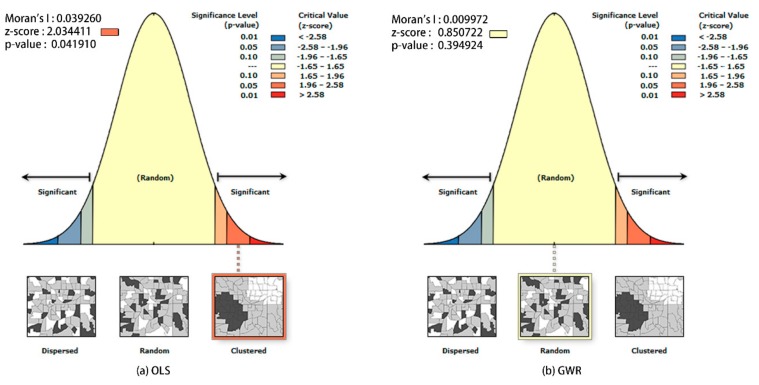
Comparison of Residual Moran’s I index for traditional least squares method (OLS) (**a**) and global weighted regression (GWR) (**b**) models.

By combining various land type areas as explanatory variables, the population quantity is used as the dependent variable, and the OLS and GWR models are constructed. Finally, the model fitting optimization is best when road and other paving are used as explanatory variables. As can be seen from Table 2, variance inflation factor (VIF) = 1.97, which is less than 7.5, indicating that there is no multiple collinearity between variables. The AIC value of the OLS model is 515.417, and that of the GWR model is 458.706. The adjusted R^2^ value of the GWR model is 0.78, which is higher than that of the OLS model, at 0.34. As shown in Figure 5, the residual Moran’s I of the OLS model is 0.04, the z-score is 2.03, and the *p*-value is 0.04, indicating that the residual error of the OLS model shows an obvious clustered pattern, and the confidence level is 95%. The Moran’s I of the GWR model is 0.01, the z-score is 0.85, the *p*-value is 0.39, and the residual error of the GWR model is in random mode. The above factors showed that the GWR model was superior to the OLS model in all aspects.

#### 4.1.2. Mapping the Population Spatial Distribution

**Figure 6:**
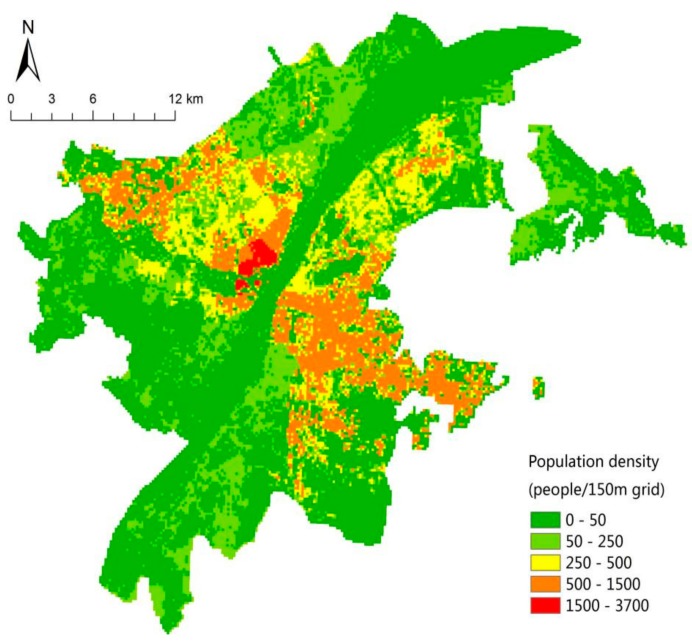
Spatialized population density map of Wuhan’s central area in 2015.

According to Formula 2, the population error of the model is tested. The total simulated population is 588,000, with an actual population of 576,000 and an overall relative error of 2.3%. The results of the error evaluation on the block scale are shown in Table 3, with an average relative error of 35.8%. Therefore, it is necessary to correct the error. On the basis of Formula 3, the corrected regression coefficient raster image can be obtained. In this study, 150 m was selected as the grid size, the road area and other paving grid images could be obtained by using the regional statistical tools, and the population spatial distribution of the 150 m grid (Figure 6) in the study area could be calculated by combining the corrected regression coefficient raster.

### 4.2. Analysis of the Spatial Accessibility of Urban Park Green Spaces

**Figure 7:**
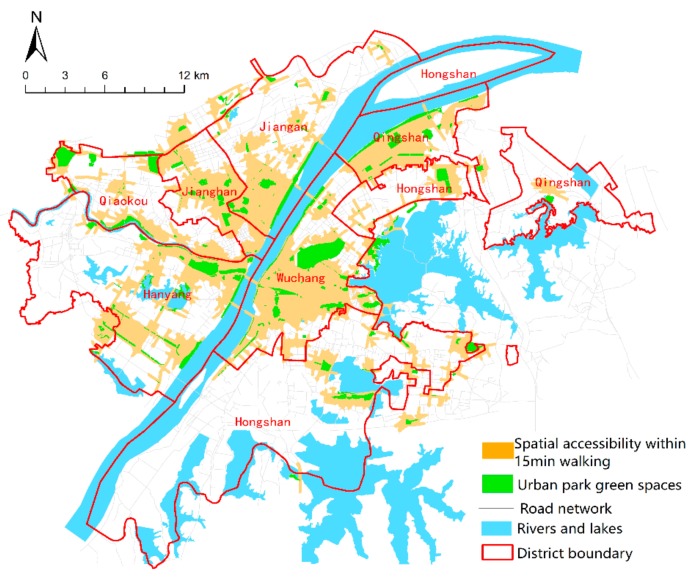
Service areas for urban park green space according to the network analysis methods.

The accessibility analysis results of urban park green space based on network analysis are shown in Figure 7: the accessible coverage area, the area within a 15 min walking range of a park, reached 161.28 km^2^, accounting for 25.8% of the total study area. In terms of spatial structure, areas with a high degree of accessibility are mainly concentrated in urban centers, including the junction of Jianghan District and Jiang’an District; most of the streets of Wuchang District, Hanyang District, and Qingshan District along the river. The green space in such areas is not only concentrated, but also the road facilities are relatively well constructed.

The areas with poor accessibility are mostly concentrated in marginal areas, mainly due to the serious shortage of green space supply in the park. However, Jiangdi Street and Baishazhou Street in the southwestern part of the study area are less accessible due to the imperfect road system. That is, the density of the road network is low, and the supply of park green space is not the main reason for this.

### 4.3. Evaluation of Green Space Equity in Urban Parks of Wuhan City

**Figure 8:**
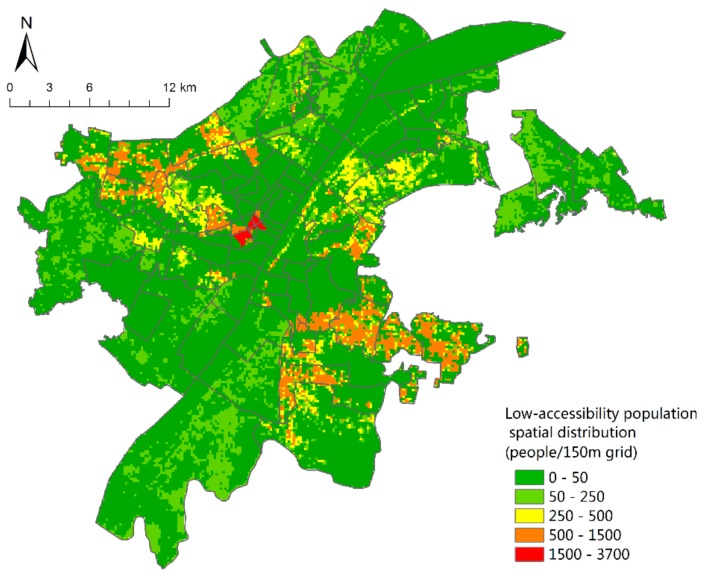
Low-accessibility population spatial distribution.

Based on the analysis of the population spatial distribution and network accessibility, spatial superposition analysis is used to obtain the results of population spatial distribution with low-accessibility (Figure 8). Also, beyond the reach of 15 min on foot, Hanzheng Street of Qiaokou District, Minyi Street, and Qianjin Street of Jianghan District and other areas of the densest population distribution comprise up to 3617 people per 150 m grid—that is, 160,755 people per km^2^—which is well above the population density of 89,200 people based on street scale statistics per km^2^ (Figure 2). In addition, Yijia Street, Changfeng Street, Gutian Street, HanJiadun Street and Shizishan Street, Luonan Street, Zhuodaoquan Street, and Guanshan Street in the northwest of the city are densely populated.

**Figure 9:**
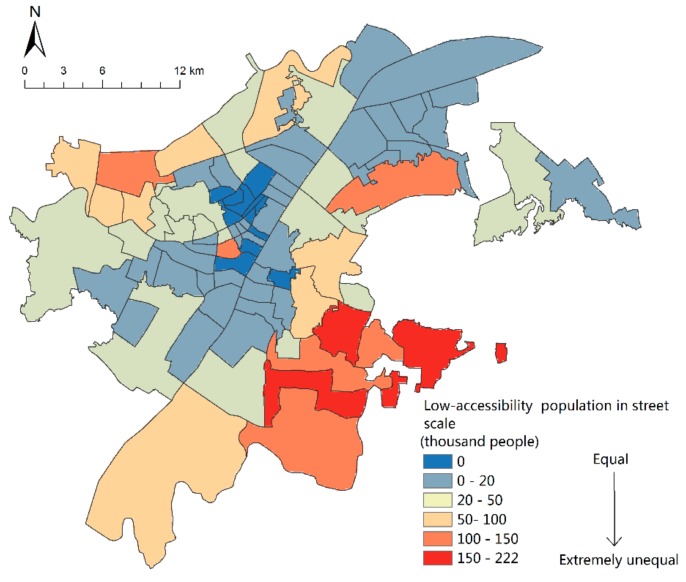
Low-accessibility population at the street scale.

According to statistics, the 15-min walking distance of Wuhan Central Park Green Space covers 3.1 million people, accounting for 54.2% of the total population. With streets as the basic unit, the total number of people outside the reach is counted, and the results (Figure 9) show that there are 12 streets at a level of equity, all of which are distributed in the center of the city. Most of these areas are close to comprehensive parks and green land along the river, with a population coverage of 100 percent. In general, except for Hanzheng Street, most of the unequal streets are located outside a 15 min walking distance, among which Hongshan Park has the worst equity. Three streets in Hongshan District are extremely unequal, namely Luonan Street, Shizishan Street, and Guanshan Street. Almost 220 thousand people on Guanshan Street are without accessibility. Hongshan District has many universities, such as Wuhan University, Huazhong University of Science and Technology, Central China Normal University, Huazhong Agricultural University, and so on. The dense distribution of colleges and universities makes the total population of the Hongshan area large. At the same time, Figure 7 also shows the scarcity of green space in the park, which leads to its inequity. In the future, Wuhan should give priority to the construction of park green space in Hongshan District to meet the recreation needs of its multitudinous users.

## 5. Discussion and Conclusions

Most of the current research on equity evaluation of green space in parks generally averages demographic data to measure the needs of users. For example, through the area ratio of the residential area and the street population data, the population of each residential area is counted and the spatial distribution of the population is calculated to evaluate the equity of the park green space [41]. There are some limitations in this method of assuming that the population is evenly distributed in residential buildings. On the basis of the study of the theory of the accessibility and equity of the green space of the park, this paper first adopted the population data spatialization method based on the land cover type to measure the demand level of the green space of the park and map the population data to the specific space unit. It is more accurate and more reliable than the traditional method (population data with the administrative division as the unit). Finally, the equity of park green space in the central city of Wuhan is evaluated quantitatively by adding the accessibility service range of park green space.

The evaluation results showed the range of park green space in the central urban area of Wuhan was within a walking distance of 15 min, accounting for 25.8% of the total study area and, covering 54.2% of the population in the study area. In addition, areas with poor equity were mainly concentrated in the urban periphery due to the scarcity of park green space resources [42]. The equity of park green space in Hongshan District was the worst, so the government should focus on the allocation of park green space in this District in the near future.

However, there are still some limitations to this article. Since the vertical distribution (high-rise buildings) is also one of the important factors affecting the spatial distribution of the population, there have been studies to explore the method of spatializing population based on building information [19]. The building data of field visits are usually suitable for small-scale research, but it is difficult to obtain accurate building height information for the whole central area of Wuhan in our research. So obtaining such detailed vertical data and higher-resolution remote sensing images are the important aspects of our future efforts. In addition to the travel mode of walking, cycling or public transport could be added in the future to improve the results of the accessibility analysis.

At present, with the development of GIS technology, the evaluation method of park green space accessibility has become more and more mature, but quantifying the public demand has been a difficult problem in the relevant fields. This paper, based on the principle of equal sharing, takes the total population as a measure of demand and aims to provide a new method for quantitative demand space quantification.

In the future, other socio-economic data, such as the distribution of the elderly, children, or low-income groups, can be used to evaluate the needs of specific groups and include them under the existing landscape equity assessment system. The evaluation results will provide more effective support for the corresponding planning strategy. In addition, only considering the accessibility level, the fairness of urban park green space can be comprehensively evaluated in terms of per capita area and park quality in the future.

## Figures and Tables

**Table 1:** Spearman correlation analysis between the population and land cover type.

Land Cover Types	Bare Land	Road	Water	Woodland	Farmland	Factory Buildings	Other Paving
Correlation coefficient	0.387 **	0.229 *	−0.072	0.220 *	0.111	0.279 **	0.529 **
Significance level	0.000	0.031	0.503	0.038	0.299	0.008	0.000

* Correlation is significant at the 0.05 level; ** Correlation is significant at the 0.01 level.

**Table 2:** Comparison of OLS and GWR model results.

	Model	OLS	GWR
	VIF	1.97 (Road)	
		1.97 (Other paving)	
Model parameter	AIC	515.417	458.706
	R^2^	0.36	0.87
	Adjusted R^2^	0.34	0.78
	Moran’s I	0.04	0.01

**Table 3:** The result of error evaluation on street scale.

Relative Error (%)		Number of Blocks
Relative error (absolute value)	≤10	20
	10~20	28
	20~30	12
	>30	29
Mean relative error	35.8	
